# Comparative Assessment of the Allergenicity of Hyaluronidases from *Polistes dominula* (Pol d 2), *Vespula vulgaris* (Ves v 2), and *Apis mellifera* Venom (Api m 2)

**DOI:** 10.3390/toxins16110498

**Published:** 2024-11-19

**Authors:** Johannes Grosch, Bernadette Eberlein, Sebastian Waldherr, Mariona Pascal, Britta Dorn, Clara San Bartolomé, Federico De La Roca Pinzón, Maximilian Schiener, Ulf Darsow, Tilo Biedermann, Jonas Lidholm, Maria Beatrice Bilò, Thilo Jakob, Carsten B. Schmidt-Weber, Simon Blank

**Affiliations:** 1 Center of Allergy and Environment (ZAUM), Technical University of Munich, School of Medicine and Health & Helmholtz Munich, German Research Center for Environmental Health, 85764 Munich, Germany; grosch.jo@gmail.com (J.G.); schiener.maximilian@gmail.com (M.S.); csweber@tum.de (C.B.S.-W.); 2 Department of Dermatology and Allergy Biederstein, School of Medicine and Health, Technical University of Munich, 80802 Munich, Germany; bernadette.eberlein@tum.de (B.E.); ulf.darsow@tum.de (U.D.); tilo.biedermann@tum.de (T.B.); 3 Immunology Department, CDB Hospital Clinic de Barcelona, Institut d’Investigacions Biomèdiques August Pi i Sunyer (IDIBAPS), Universitat de Barcelona, 08007 Barcelona, Spain; mpascal@clinic.cat; 4 Spanish Network for Allergy-RETIC de Asma, Reacciones Adversas y Alérgicas (ARADYAL), 28040 Madrid, Spain; 5 Experimental Dermatology and Allergy Research Group, Department of Dermatology and Allergology, Justus-Liebig-University Gießen, 35392 Gießen, Germany; britta.dorn@derma.med.uni-giessen.de (B.D.); thilo.jakob@derma.med.uni-giessen.de (T.J.); 6 Immunology Department, CDB Hospital Clinic de Barcelona, Universitat de Barcelona, 08007 Barcelona, Spain; clara.sbb3@gmail.com; 7 Allergy Section, Hospital Clinic de Barcelona, Clinical Medical Group, 08036 Barcelona, Spain; delaroca.federico@gmail.com; 8 Thermo Fisher Scientific, 75450 Uppsala, Sweden; jonas.lidholm@thermofisher.com; 9 Department of Clinical and Molecular Sciences, Polytechnic University of Marche, 60121 Ancona, Italy; m.b.bilo@staff.univpm.it; 10 Allergy Unit, Department of Internal Medicine, University Hospital of Ancona, 60126 Ancona, Italy; 11 Istituto di Ricovero e Cura a Carattere Scientifico (IRCCS), Istituto Nazionale di Riposo e Cura per Anziani (INCRA), 60121 Ancona, Italy

**Keywords:** allergen cross-reactivity, Api m 2, *Apis mellifera*, hyaluronidases, Hymenoptera venom allergy, Pol d 2, *Polistes dominula*, Ves v 2, *Vespula vulgaris*

## Abstract

Sensitization to cross-reactive allergens complicates identifying the culprit insect in Hymenoptera venom allergy via diagnostic tests. This study evaluates sensitization to hyaluronidases (Api m 2 from honey bee (*Apis mellifera*) venom, HBV; Pol d 2 from European paper wasp (*Polistes dominula*) venom, PDV; and Ves v 2.0101 and Ves v 2.0201 from yellow jacket (*Vespula vulgaris*) venom, YJV) and their cross-reactivity in allergic patients from Italy, Spain, and Germany using ImmunoCAPs, ELISA, and basophil activation tests. Sensitization rates were 45% for Api m 2 in HBV-allergic subjects, 25% for Pol d 2 in PDV-allergic individuals, and 20% and 10% for Ves v 2.0201 and Ves v 2.0101 in YJV-allergic patients, respectively. Patients primarily sensitized to Api m 2 showed minimal cross-reactivity to vespid hyaluronidases, whereas those primarily sensitized to Pol d 2 or Ves v 2.0201 exhibited IgE reactivity to Api m 2. Neither Pol d 2 nor Ves v 2.0201 triggered basophil activation. Cross-reactivity of Api m 2, Pol d 2, and Ves v 2.0201 depends on the primary sensitizing venom. Sensitization to Pol d 2 and Ves v 2.0201 remains below 25%, yet these patients may exhibit cross-reactivity to Api m 2. Conversely, HBV-allergic patients sensitized to Api m 2 show minimal reactivity to Pol d 2 or Ves v 2.0201.

## 1. Introduction

Hyaluronidases, ubiquitous components of various species’ venoms, span across taxonomic orders, including Hymenoptera (bees, wasps, hornets, and ants) [1,2,3], snakes, spiders, and even the platypus [4,5,6]. They play a crucial role in allergic reactions triggered by Hymenoptera stings. Allergic reactions following Hymenoptera stings in Europe are primarily induced by venoms of the Hymenoptera species *Apis mellifera* (honey bee) and *Vespula* spp. (yellow jacket), with Southern Europe additionally facing reactions related to *Polistes dominula* (European paper wasp), an invasive species spreading northward [7,8,9]. *P. dominula* has also conquered new habitats around the globe, such as Northern America and South Africa [10,11], underscoring the global importance of understanding their venom components. Another highly invasive Hymenoptera species, the yellow-legged or Asian hornet (*Vespa velutina nigrithorax*), is progressively establishing itself across Europe, even though its natural habitat is in tropical regions of Southeast Asia. In areas of Europe where it has become established, *Vespa velutina nigrithorax* has become a notable cause of Hymenoptera-induced anaphylaxis [12].

The key hyaluronidases of interest to a wider population include Api m 2 from honey bee venom (HBV), Ves v 2.0101 and Ves v 2.0201 from yellow jacket venom (YJV), and Pol d 2 from *P. dominula* venom (PDV). Sensitization rates vary among patient populations, with Api m 2 sensitization ranging from 28% to 60% [13,14,15,16,17]. Currently, routine diagnostic tools focus on Api m 2, as other Hymenoptera hyaluronidases are less investigated [18]. Experimental studies suggest sensitization rates of approximately 5% for Ves v 2.0101 and 20–28% for Ves v 2.0201 [13,19,20]. Only limited data are available regarding the allergenic potential of Pol d 2 [1] and the cross-reactivity among Hymenoptera venom hyaluronidases, highlighting gaps in the understanding essential for tailored treatment strategies.

Beyond their role in allergies, hyaluronidases also serve important functions within their venom, cleaving hyaluronan, a crucial component of connective tissue. Hyaluronan is composed of disaccharide subunits consisting of *N*-acetyl-β-d-glucosamine and β-d-glucuronic acid, which are linked via alternating β-1,3 and β-1,4 glycosidic bonds and together build the repetitive structure of hyaluronan in the extracellular matrix. The proteins of Enzyme Commission number 3.2.1.35, the so-called hyaluronoglucosidases, e.g., Api m 2, hydrolyze the β-1,4 linkage of hyaluronan, rendering small and non-functioning subunits [21]. While hyaluronidases do not have a direct toxic effect, they intensify the effect of other venom proteins. The hydrolysis of hyaluronan and chondroitin A and C supports the diffusion of the venom through tissues adjacent to the puncture site and the distribution of the venom via blood vessels in the victim [22,23]. Because of this spreading function, hyaluronidases can play a significant role in local and systemic venom reactions without imparting a direct venomous effect [5,24]. Interestingly, Ves v 2.0201 represents an inactive variant that carries a point mutation at the enzyme’s active site and seems to be the dominant isoform in YJV [25].

To shed light on the prevalence of sensitization among Hymenoptera venom-allergic individuals and to evaluate the true, cross-reactive carbohydrate determinant (CCD)-independent cross-reactivity of pertinent venom hyaluronidases, Api m 2, Pol d 2, Ves v 2.0101, and Ves v 2.0201 were recombinantly produced in insect cells. The investigation of sensitization to Ves v 2.0101 was performed using experimental ImmunoCAPs and included a cohort of YJV- and/or HBV-allergic patients. Furthermore, we assessed sensitization and cross-reactivity of the less explored hyaluronidases Ves v 2.0201 and Pol d 2 through ELISA, comparing them with Api m 2. The capacity of these hyaluronidases to trigger effector cells was examined ex vivo using basophil activation tests (BAT).

## 2. Results

### 2.1. Structural Features of the Investigated Hymenoptera Hyaluronidases

**Figure 1:**
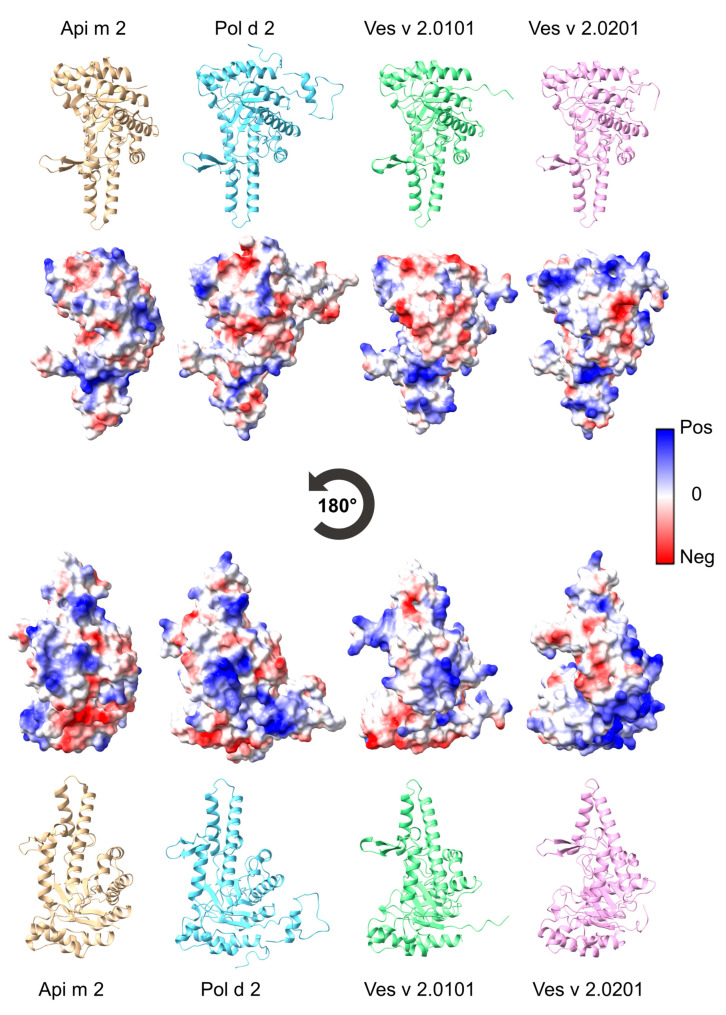
Structural features of venom hyaluronidases. Ribbon diagrams and the surface coulombic electrostatic potential (ESP) of Api m 2, Pol d 2, Ves v 2.0101, and Ves v 2.0201 are displayed. Positive ESP areas are colored blue, and negative ESP areas are red. The structures of Api m 2 (PDB: 1FCU) and Ves v 2.0101 (PDB: 2ATM) were solved by crystallography, and those of Pol d 2 and Ves v 2.0201 were generated by structural modeling.

**Figure 2:**
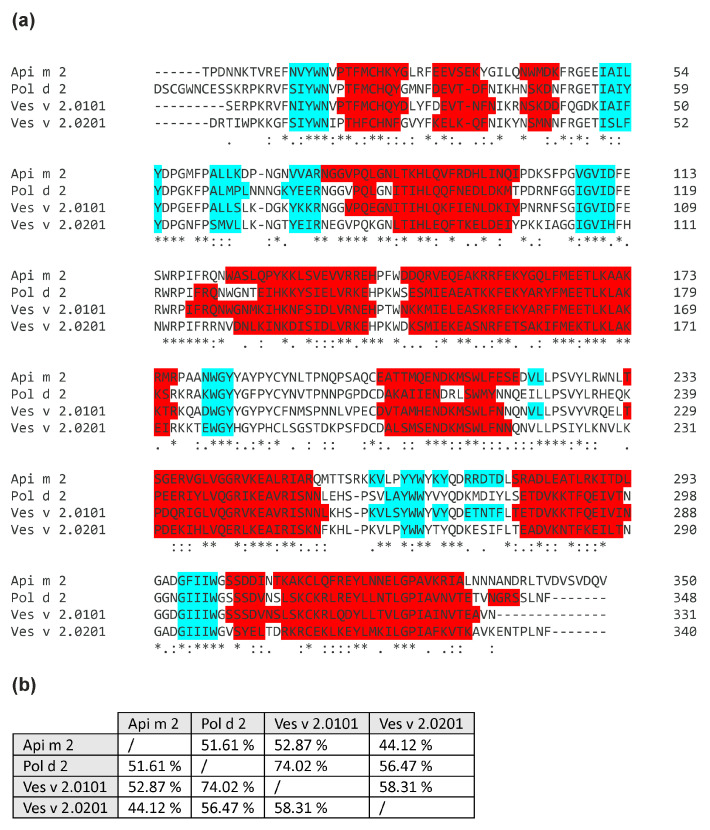
Sequence alignment and identity of venom hyaluronidases. (**a**) Alignment of the mature sequences of the investigated venom hyaluronidases. Turquoise and red boxes indicate the positions of β-strand and α-helices, respectively. Asterisks, colons, and periods indicate identical, conserved, and semi-conserved residues, respectively. (**b**) Percent identity between the different hyaluronidase allergens.

Figure 1 shows the tertiary structures of the hyaluronidases Api m 2 (PDB: 1FCU) [26], Pol d 2, Ves v 2.0101 (PDB: 2ATM) [27], and Ves v 2.0201. Pol d 2 and Ves v 2.0201 structures were modeled based on the structure of Ves v 2.0101 with a confidence of 100% for both and sequence identity of 74% and 59%, respectively (Figure 2). Despite the intermediate sequence identity of 44–74% of Api m 2, Pol d 2, Ves v 2.0101, and Ves v 2.0201 (Figure 2), the tertiary structure of the hyaluronidases is highly conserved.

As the binding of conformation-dependent epitopes is based not only on the three-dimensional structure of a protein but also on its surface charge, the coulombic electrostatic potential (ESP) was calculated and added to Figure 1. While no apparent difference can be described solely based on tertiary structure, the surface charges of the hyaluronidases of interest draw a different picture. All four proteins have unique areas of ESP surface charge, creating potential exclusive allergenic epitopes while maintaining enough similarities to their respective homologous proteins to exhibit cross-reactivity.

### 2.2. sIgE Sensitization of Allergic Patients to Ves v 2.0101

**Figure 3:**
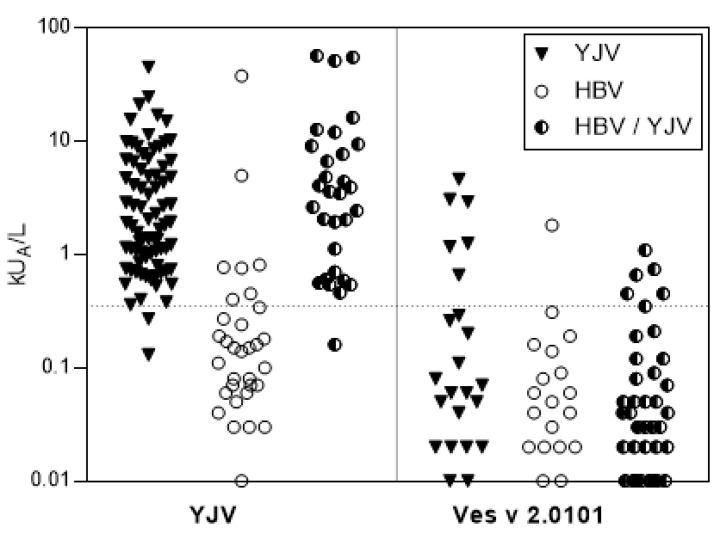
sIgE sensitization to yellow jacket venom Ves v 2.0101. Prevalence of sensitization in HBV-, YJV-, and HBV/YJV-reactive patients to whole YJV and Ves v 2.0101 as measured by ImmunoCAP. A dotted line indicates the 0.35 kU_A_/L cut-off. HBV, honey bee venom; YJV, yellow jacket venom.

The sensitization rate to the hyaluronidase Ves v 2.0101 from YJV was addressed using experimental ImmunoCAPs with the recombinant CCD-free allergen. Data were collected for patients with a positive cutaneous test for HBV, YJV, or HBV and YJV (HBV/YJV) (Figure 3). In the YJV-reactive group, 7.7% (6/78) of patients were positive for Ves v 2.0101 (sIgE ≥ 0.35 kU_A_/L) compared with a single patient (1/35) in the HBV-reactive group who was also Api m 2-positive. Most reactivity, 20% (6/30), was measured in the double-reactive group. Due to the generally low sensitization rates against Ves v 2.0101, further work was focused on the remaining potentially allergy-relevant venom hyaluronidases Api m 2, Pol d 2, and Ves v 2.0201.

### 2.3. Characterization of Recombinant Hyaluronidases

**Figure 4:**
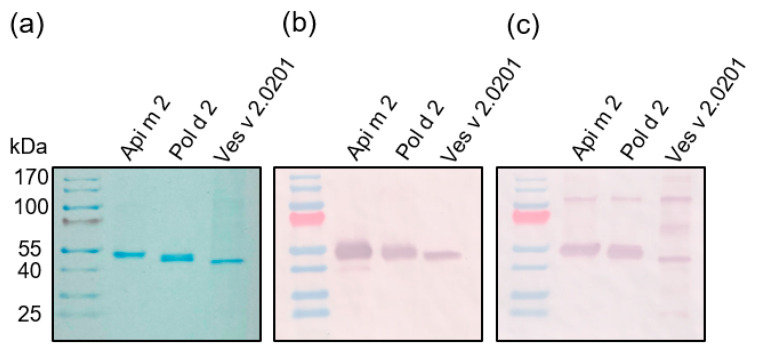
Recombinant expression and characterization of venom hyaluronidases. SDS-PAGE and immunoblot of recombinant Api m 2, Pol d 2, and Ves v 2.0201 visualized by either (**a**) Coomassie blue staining or (**b**) anti-V5 epitope antibody and (**c**) *Galanthus nivalis* agglutinin (GNA).

Using Sf9 insect cells, the hyaluronidases were recombinantly expressed devoid of CCDs but with the remaining *N*-glycosylations intact. After purification, the proteins were analyzed by SDS-PAGE and either stained with Coomassie blue or immobilized onto nitrocellulose membranes. Western blots were performed with an anti-V5 antibody directed against the co-expressed V5-tag and *Galanthus nivalis* agglutinin (GNA) to detect N-glycosylation (Figure 4).

The predicted molecular weights (MW) for Api m 2, Pol d 2, and Ves v 2.0201 are 42, 41, and 40 kDa, respectively. When analyzed by reducing SDS-PAGE and staining with Coomassie blue or detection with anti-V5-tag antibody, the three hyaluronidases migrated between 40 and 55 kDa, roughly confirming their predicted MWs (Figure 4a,b). The GNA blots show bands for Api m 2, Pol d 2, and Ves v 2.0201 (Figure 4c), therefore verifying the in silico-predicted number of *N*-glycosylation sites (predicted sites: Api m 2: 3; Pol d 2: 2; Ves v 2.0201: 2).

### 2.4. sIgE Sensitization of Allergic Patients to Api m 2, Pol d 2, and Ves v 2.0201

sIgE sensitization of HBV-, PDV-, and YJV-allergic patients was assessed in vitro by ELISA using recombinant, CCD-free Api m 2, Pol d 2, and Ves v 2.0201. Of the 95 patients included, 29 were mono-reactive in the intradermal test with HBV, 16 with PDV, and 18 with YJV. Further, 24 patients were double-reactive with PDV and YJV, and eight were triple-reactive with HBV, PDV, and YJV.

Api m 2 was recognized by 45% (13/29) of HBV, 25% (4/16) of PDV, and 20% (3/18) of YJV mono-reactive; 15% (4/24) of PDV/YJV double-reactive; and 25% (2/8) of HBV/PDV/YJV triple-reactive patients. The sensitization rates against Pol d 2 were approximately 15% (4/29) in HBV-, 25% (4/16) in PDV-, 20% (3/18) in YJV-, 15% (4/24) in PDV/YJV-, and 10% (1/8) in HBV/PDV/YJV-reactive patients. The hyaluronidase Ves v 2.0201 from YJV showed overall slightly lower sensitization rates in the tested patient populations: 20% (5/29) in HBV, 10% (1/16) in PDV, and 20% (3/18) in YJV mono-reactive; 5% (2/24) in PDV/YJV double-reactive; and 25% (2/8) in HBV/PDV/YJV triple-reactive patients (Table 1).

**Figure 5:**
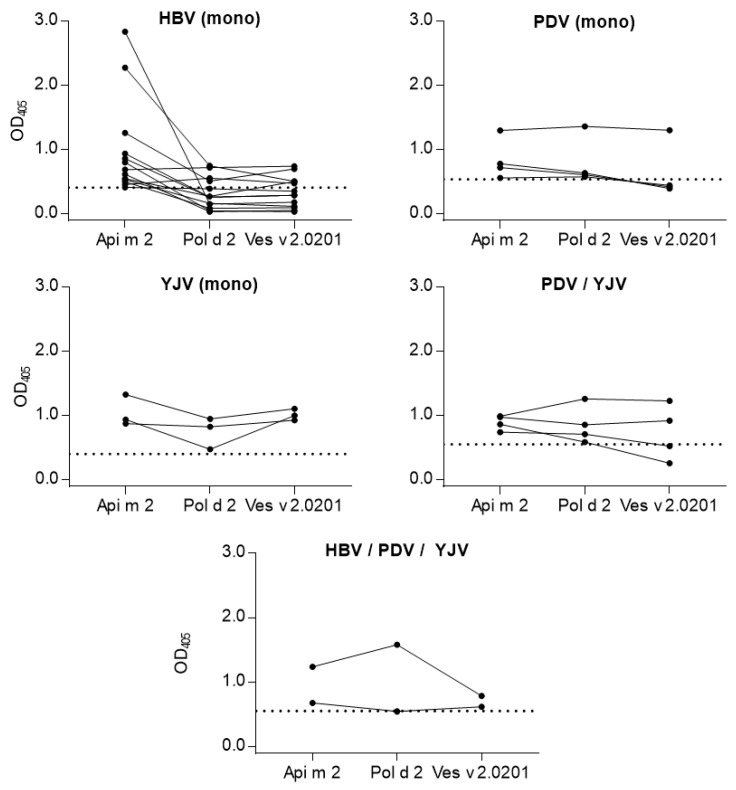
Comparison of sIgE reactivity to venom hyaluronidases. Cross-reactivity of Api m 2, Pol d 2, and Ves v 2.0201 was assessed in HBV (mono)-, PDV (mono)-, YJV (mono)-, PDV/YJV double-, and HBV/PDV/YJV triple-reactive patients. HBV, honey bee venom; mono, mono-reactive in skin test; PDV, *Polistes dominula* venom; YJV, yellow jacket venom.

To illustrate the putative cross-reactivity of the hyaluronidases, patients negative to all tested hyaluronidases were excluded, and the reactivities for each population were merged into one graph. In Figure 5, dots representing an individual patient’s reactivity to either Api m 2, Pol d 2, or Ves v 2.0201 were connected to allow a quick assessment of the patient’s capability to bind more than one hyaluronidase homolog. Four of the twelve HBV-reactive patients sensitized to Api m 2 had measurable IgE against Pol d 2 and three to Ves v 2.0201. All three PDV-reactive patients sensitized to Pol d 2 were also able to bind Api m 2, but only one had detectable IgE against Ves v 2.0201. The three patients reactive to YJV who were sensitized to Ves v 2.0201 also reacted to Pol d 2 and Api m 2 in vitro. Half of the PDV/YJV double-reactive patients above the cut-off reacted to Api m 2, Pol d 2, and Ves v 2.0201. The other half was sensitized to Api m 2 and Pol d 2 but not Ves v 2.0201. All triple-reactive patients with at least one sensitization to the tested hyaluronidases reacted to Api m 2, Pol d 2, and Ves v 2.0201.

### 2.5. Reactivity of Api m 2, Pol d 2, and Ves v 2.0201 in Basophil Activation Test

Basophil activation tests were performed with patients allergic to HBV, PDV, YJV, or a combination thereof. Basophils were treated with increasing doses of recombinant hyaluronidases, and the upregulation of CD63 on the cell surface was measured. Patients were enrolled in the study in the greater Munich (Germany, *n* = 17, MUC) or Barcelona (Spain, n = 12, BCN) areas. As patients were recruited from clinical routine, and assessing sensitization against PDV is not part of the general diagnostic algorithm in Germany, sensitization against PDV cannot be excluded for those patients. Patients with HBV sensitization were not representative of the general HBV-allergic population since patients with sensitization to Api m 2 were preferably included.

**Figure 6:**
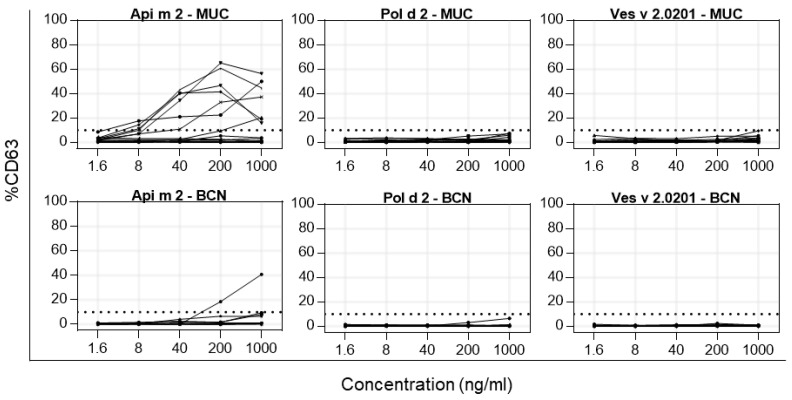
Basophil activation tests with recombinant venom hyaluronidases. Human basophils from Hymenoptera venom-allergic patients from Munich, Germany (MUC), and Barcelona, Spain (BCN), were exposed to different concentrations of recombinant Api m 2, Pol d 2, or Ves v 2.0201, and the increase in CD63 on the cell surface was measured. Activation is shown as a percentage increase of CD63^+^ out of total basophilic cells. Cut-off (dotted line) is at 10% of CD63 increase.

Seven of the seventeen MUC patients showed basophil activation upon stimulation with Api m 2 (Figure 6). However, none reacted to Pol d 2 or Ves v 2.0201. Basophil activation with Api m 2 was further seen in one BCN patient (Figure 6). Although clear sensitization to PDV and/or YJV was demonstrated in vitro for nine out of the twelve BCN patients, no CD63 upregulation was seen after stimulation with either Pol d 2 or Ves v 2.0201.

## 3. Discussion

The Hymenoptera venom hyaluronidases Api m 2, Pol d 2, Ves v 2.0101, and Ves v 2.0201 were analyzed using a variety of in silico tools, allowing a comparison of primary, secondary, and tertiary structures, including surface charge. Sensitization to Ves v 2.0101 was tested using experimental ImmunoCAPs. Furthermore, Api m 2, Pol d 2, and Ves v 2.0201 were recombinantly produced in *Spodoptera frugiperda* (Sf9) insect cells. IgE antibodies against α-1,3-core-fucosylation of Hymenopteran carbohydrate structures are frequently found in venom-allergic patients [28]. However, no clinical relevance has been established so far [29]. This specific carbohydrate linkage, also known as cross-creative carbohydrate determinant (CCD), is one major reason for IgE cross-reactivity between venoms of different Hymenoptera species. This, however, does not represent a clinically relevant sensitization. Sf9 cells add carbohydrate modifications to the recombinant proteins, which can be beneficial for proper folding. However, the attached carbohydrate structure lacks the α-1,3-core-fucosylation, which is the molecular basis for CCD reactivity [19]. This allowed a direct comparison of true protein allergenicity without the interfering influence of CCDs, which are known to distort the prevalence of sensitization in Hymenoptera venom-allergic patients [28]. The full-length recombinant proteins were used in ELISA to determine the presence of sIgE in patient sera and in BAT to evaluate their ability to activate effector cells of patients sensitized to HBV, PDV, YJV, PDV/YJV, and HBV/PDV/YJV.

Despite the intermediate levels of sequence identity of 44–74% between Api m 2, Pol d 2, Ves v 2.0101, and Ves v 2.0201, the secondary and tertiary structures seem to be conserved, and conformational, allergenic epitopes mediating cross-reactivity between the species’ venoms are probably maintained. While surface charges of Api m 2, Pol d 2, Ves v 2.0101, and Ves v 2.0201 differ in certain areas, others share enough similarities for cross-reactivity based on ESP and structure.

Structural similarities, particularly in the context of conserved conformational epitopes, likely contribute to cross-reactivity by promoting immune recognition across different allergens. Conformational epitopes—three-dimensional structures formed by amino acid sequences brought into proximity through protein folding—play a significant role in IgE binding. Because IgE antibodies recognize these spatial arrangements, structural similarities between allergens such as Api m 2, Pol d 2, and Ves v 2.0201 may lead to cross-reactivity, even when the sequence homology is limited. This underlines the importance of considering both linear and conformational epitope analysis in studies of allergen cross-reactivity to improve clinical outcomes.

The experimental ImmunoCAPs performed with Ves v 2.0101 confirmed its role as a minor allergen in YJV- and HBV-allergic patients (<10% of patients sensitized with sIgE titers ≥0.35 kU/L). Relevant sensitization rates were only measured for YJV/HBV double skin-reactive patients (~20%), which aligns with already published data [19,20].

After recombinant production and purification, the predicted MWs of 42 (Api m 2), 41 (Pol d 2), and 40 (Ves v 2.0201) kDa were confirmed by SDS-PAGE. However, a slightly higher MW was observed, probably due to N-glycosylation on the hyaluronidases. N-glycosylation was confirmed by detecting the hyaluronidase bands with the lectin GNA.

The recombinant full-length proteins were tested in ELISA with 95 patients recruited in Italy who were skin-reactive to HBV (29), PDV (16), YJV (18), PDV/YJV (24), and HBV/PDV/YJV (8). The assessed prevalence of sensitization in these patients confirmed the importance of Api m 2 in HBV-reactive individuals (45% sensitized). Additionally, it indicates that true sensitization to Pol d 2 and Ves v 2.0201 is less prevalent. Furthermore, 20% of YJV-reactive patients reacted to Ves v 2.0201. The prevalence of sIgE to Api m 2 in HBV- and Ves v 2.0201 in YJV-reactive patients was consistent with previously published data [14,19,20]. In PDV mono-reactive patients, 25% had detectable sIgE levels directed against Pol d 2. Around 15% of PDV/YJV double-reactive patients reacted to Pol d 2, while only 5% reacted to Ves v 2.0201. The obtained in vitro data are in line with published sensitization rates for Api m 2 and Ves v 2.0201 in HBV- and YJV-allergic patients and adds a further piece to the puzzle by describing sensitization to Api m 2 and Ves v 2.0201 in PDV- and PDV/YJV-allergic patients. The recently identified Pol d 2 showed sensitization rates comparable to those of Ves v 2.0201, in the range of 15 to 25%, depending on the population tested.

Including different Hymenoptera venom-allergic patient groups allowed us to assess the putative cross-reactivities of venom hyaluronidases. The majority of HBV skin-reactive patients sensitized to Api m 2 did not react to Pol d 2 or Ves v 2.0201. However, four HBV-allergic patients tested positive for Pol d 2 and Ves v 2.0201. It remains speculative whether this cross-sensitization renders patients susceptible to systemic reactions when exposed to hyaluronidases from other species. Factors that might influence the clinical relevance of cross-reactivity include IgE antibody affinity and density of cross-reactive epitopes on the homologous allergens.

All PDV-allergic patients sensitized to Pol d 2 also tested positive against Api m 2. Only one reacted to Ves v 2.0201, confirming Pol d 2 as probably cross-reactive with Api m 2 and, to a lesser degree, to Ves v 2.0201. Patients mono-reactive to YJV who had detectable sIgE levels against Ves v 2.0201 were all reactive to Api m 2 and Pol d 2, showing the potential cross-reactivity of Ves v 2.0201. The high positive rate for Api m 2 in patients sensitized to Pol d 2 or Ves v 2.0201 in PDV/YJV-allergic individuals (4/4 and 2/4) shows the extensive cross-reactivity of wasp and honey bee hyaluronidases in this patient group. This is in line with prior work showing that 10–15% of people allergic to YJV have IgE against Ves v 2.0101 or Ves v 2.0201 (no discrimination is made). Half of these patients showed peptide-specific cross-reactivity with Api m 2 [20].

The data indicate that the initially sensitizing venom has an effect on the cross-reactivity of venom hyaluronidases. While there is a limited likelihood for primarily Api m 2-sensitized patients to react to Pol d 2 and Ves v 2.0201, virtually all Pol d 2- and Ves v 2.0201-sensitized patients react to Api m 2. Ves v 2.0201-sensitized patients can further recognize Pol d 2, while the chance for primarily Pol d 2-sensitized patients to react to Ves v 2.0201 appears to be less pronounced. This might be explained by allergenic epitopes present on the primarily sensitizing hyaluronidases, which are lacking in the putative cross-reactive homologs.

The observed cross-reactivity among these allergens may have implications for both diagnosis and treatment, particularly for patients at high risk of systemic reactions. In diagnostics, cross-reactivity can complicate the identification of primary sensitizing allergens, potentially leading to misdiagnosis or over-diagnosis if clinicians rely on extract-based IgE testing alone. To address this, a CRD approach may improve diagnostic accuracy by differentiating between genuine sensitization to one allergen and cross-reactive responses due to protein similarities.

Measuring Api m 2 as part of the diagnostic work up may add clinical value in patients suspected of HVA. A positive result in liaison with sensitization to HBV species-specific markers (e.g., Api m 1 and Api m 10), may explain positive YJV and/or PDV extract results in the absence of YJV and/or PDV species-specific markers (e.g., Ves v 5 and Pol d 5). This allows an increased resolution, enabling a patient-tailored treatment strategy.

On the other hand, Pol d 2 and Ves v 2.0201 may be of limited value considering (a) the low sensitization rates in YJV and/or PDV allergic patients and (b) the observed cross-reactivity with Api m 2. Given the positive Api m 2 results in YJV and/or PDV allergic patients with sensitization to the respective species-specific hyaluronidase (Ves v 2.0201 or Pol d 2), Api m 2 may suffice as a marker allergen for hyaluronidase sensitization.

From a therapeutic standpoint, understanding cross-reactivity is crucial in selecting the most appropriate extract for immunotherapy. An increased diagnostic resolution may help to identify the right AIT while reducing the cost and impact on quality of life associated with unnecessary treatments. Furthermore, tailoring treatment based on CRD could enhance the safety profile and efficacy of allergen-specific immunotherapy for some patients.

We recognize that the relatively low sensitization rates to Pol d 2 and Ves v 2.0201 in our study may affect the statistical power for detecting cross-reactive patterns. However, our findings still provide valuable initial insights into the potential cross-reactivity between Api m 2, Pol d 2, and Ves v 2.0201. These results contribute to the growing body of knowledge on Hymenoptera venom allergen sensitization. However, to strengthen and expand upon these findings, further studies with larger patient cohorts will be beneficial. Ideally, future research would involve a multicenter study across diverse geographic regions, which would not only enhance statistical power but also capture a broader range of sensitization profiles. A standardized component-resolved diagnostic (CRD) approach across sites could further improve consistency and comparability of results, enabling a more comprehensive understanding of cross-reactivity patterns. This would help determine the applicability of our observations on a larger scale.

To assess the relevance of hyaluronidase sensitization of Api m 2, Pol d 2, and Ves v 2.0201 in cellular testing, BATs were performed with Hymenoptera venom-allergic patients from Barcelona (Spain) and Munich (Germany). In Germany, Api m 2 sensitization was an inclusion criterion for the patients. Upon stimulation with Api m 2, a clear upregulation of CD63 on basophilic cells was observed in 8 out of all 29 patients, translating to an overall response rate of about 28% in Hymenoptera venom-allergic patients. However, none of the patients—including those allergic to PDV and/or YJV—reacted to stimulation with Pol d 2 or Ves v 2.0201. While this may imply that peptide-mediated sensitization to Pol d 2 or Ves v 2.0201 is of low clinical relevance, individual patients might still be susceptible to their allergenic capacities with a potentially systemic reaction. The discrepancy between IgE reactivity and the absence of response in BAT assays is intriguing and warrants further investigation. It is known that BAT often has a higher informative value for assessing clinical relevance compared to sIgE determination [30]. Consequently, the lack of response to Pol d 2 and Ves v 2.0201 in sensitized patients might reflect a limited clinical relevance of these allergens. Another possible explanation is that, in their present form, the epitopes of these allergens may not effectively induce cross-linking of IgE receptors on basophils, thereby failing to trigger cellular activation. Further studies are needed to explore these mechanisms and their implications for understanding allergen-specific reactivity and potential clinical impact.

## 4. Conclusions

In conclusion, our study highlights Api m 2 as a key allergen in HBV allergy, which is capable of activating basophils in susceptible patients and serving as a critical marker for primary HBV sensitization. While Pol d 2, Ves v 2.0101, and Ves v 2.0201 appear to play a minor role as allergens, their relevance cannot be completely ruled out due to occasional cases of primary sensitization. Emphasizing the limited cross-reactivity of Api m 2 with vespid homologs in the absence of CCDs underscores its value in distinguishing HBV allergy. To enhance diagnostic accuracy, the development and availability of commercial sIgE assays for Ves v 2 and Pol d 2 would be valuable. Such assays could improve patient care by enabling more precise identification of multi-sensitized individuals, ultimately allowing for more tailored treatment approaches and reducing unnecessary interventions in clinical practice.

## 5. Materials and Methods

### 5.1. In Silico Analysis

The hyaluronidases were bioinformatically analyzed regarding their molecular properties using ProtParam [31]. SignalP4.0 [32] was used to predict the presence and length of signal sequences. Sequence similarity was assessed by Clustal Omega [33]. Potential glycosylation of the proteins was determined by NetNGlyc 1.0 Server. Pol d 2 and Ves v 2.0201 were structurally modeled based on Ves v 2.0101 (2 ATM) [27] by Phyre^2^ [34] with 100% confidence. Three-dimensional protein structures were compared with ChimeraX v1.3 [35]. Graphs and statistical analyses were generated using GraphPad Prism 7.04 (GraphPad Software, San Diego, CA, USA).

### 5.2. Recombinant Allergen Production

Api m 2, Ves v 2.0101, and Ves v 2.0201 were cloned by Seisman et al. [19]. The Pol d 2 encoding open reading frame was amplified from *P. dominula* venom gland cDNA omitting the signal peptide. A 10-fold His-tag and a V5-tag were added to the construct for purification and unambiguous identification. The amplicon and pAcGP67B-vector (BD Pharmingen, Heidelberg, Germany) were digested with *Xba*I/*Not*I and ligated. Recombinant baculovirus was obtained by co-transfecting 1 × 10^6^ adherent Sf9 (*Spodoptera frugiperdia*) cells (Thermo Fisher Scientific, Schwerte, Germany) with ProGreen^TM^-Baculovirus DNA (AB Vector, San Diego, CA, USA) and pAcGP67B-vector carrying the Pol d 2-coding gene. High titers of baculovirus were generated by infecting 24 × 10^6^ adherent Sf9 cells, incubating for five days at 27 °C, and harvesting the supernatant by centrifugation at 3000 relative centrifugal force (rcf) for 5 min.

Recombinant full-length hyaluronidases were produced with a baculovirus-mediated insect cell expression system as described earlier [19]. In brief, wildtype Sf9 cells were cultivated in suspension at 27 °C using Insect-XPRESS protein-free medium (Lonza, Basel, Switzerland) supplemented with l-glutamine and 10 µg/mL gentamycin sulfate solution (Carl Roth, Karlsruhe, Germany). A suspension culture of 400 mL with a cell density of 1.5 × 10^6^ cells/mL was inoculated with 1 mL of high-titer virus and then incubated for 72 h at 27 °C. Recombinant proteins were isolated through metal affinity chromatography using a nickel-chelating matrix (HisTrap excel, GE Healthcare Life Sciences, Freiburg, Germany) on an ÄKTA™ pure system (GE Healthcare Life Sciences, Freiburg, Germany). After application of the supernatant, the columns were rinsed with 45 mM imidazole, and His-tagged proteins were subsequently eluted using 300 mM imidazole. The eluent was then concentrated and diafiltrated (PBS, pH 7.4) through 10 kDa cut-off Amicon^®^ Ultra centrifugal filters (Merck, Darmstadt, Germany).

### 5.3. SDS-PAGE and Western Blots

Recombinant proteins were separated by molecular weight using freshly prepared 10% Tris-Tricine gels, followed by visualization through Coomassie blue staining. For Western blotting, proteins were transferred onto nitrocellulose membranes, which were then blocked for 1 h at room temperature with either 4% (*w*/*v*) non-fat dried milk powder (AppliChem, Darmstadt, Germany) in PBS or 1% (*w*/*v*) polyvinylpyrrolidone in PBS. The immobilized proteins were probed with a mouse monoclonal anti-V5 antibody (0.2 µg/mL, Thermo Fisher Scientific, Schwerte, Germany) or biotinylated *Galanthus nivalis* agglutinin (10 µg/mL, Vector Laboratories, Peterborough, UK), followed by anti-mouse IgG (0.4 µg/mL) or ExtrAvidin (1:20,000) conjugated to alkaline phosphatase. Visualization of the complexes was achieved using nitrotetrazolium blue chloride and 5-bromo-4-chloro-3-indolyl phosphate (AppliChem, Darmstadt, Germany) as substrates.

### 5.4. Patientsh

For experimental ImmunoCAP tests with Ves v 2.0101, 35 HBV, 78 YJV, and 30 HBV/YJV skin-reactive patients with a history of systemic sting reactions were recruited in Germany (Freiburg). To assess cross-reactivity and sensitization to Api m 2, Pol d 2, and Ves v 2.0201, 17 patients sensitized to HBV, PDV, YJV, or a combination thereof were recruited out of clinical routine in Germany (Munich), 95 in Italy (Ancona) and 12 in Spain (Barcelona). Diagnostic criteria for inclusion were a thorough and positive clinical history of an allergic reaction following a Hymenoptera sting, positive intradermal skin test, and/or specific IgE (sIgE) levels as measured by ImmunoCAP system (Thermo Fisher Scientific, Uppsala, Sweden) to HBV (i1), PDV (i77), and/or YJV (i3) and/or allergen components (Api m 1 (i208), Ves v 5 (i209), Pol d 5 (i210), Ves v 1 (i211), Api m 2 (i214), Api m 3 (i215), Api m 5 (i216), and Api m 10 (i217)) or IMMULITE 2000 immunoassay (Siemens Healthineers, Erlangen, Germany) for HBV (i1) and/or YJV (i3) and/or Api m 2 (A46). For assessment of reactivity in BAT in Germany, patients with an allergy to HBV or YJV and sensitization to Api m 2 were included. For these patients, PDV was not tested in skin tests, as this is not part of the clinical routine in Germany. Table S1 shows the clinical data for all participating patients.

The assignment of patients to HBV-, PDV-, YJV-, HBV/YJV-, PDV/YJV-, or HBV/PDV/YJV-reactive patient groups was based on the skin reactivity of each patient to the venoms in question.

Written informed consent was obtained from all participants prior to their enrollment in the study. The study adhered to the principles outlined in the Declaration of Helsinki and received approval from the following ethics committees: the Ethics Committee of the Faculty of Medicine at the Technical University of Munich (5478/12, approval date: 9 October 2012), the Ethics Committee of Albert-Ludwigs-Universität Freiburg (390/12, approval date: 8 October 2012), the Comitè Ètic d’Investigació Clínica at Hospital Clínic de Barcelona (HCB/20196/08290361, approval date: 24 January 2020), and the Ethics Committee of the University Hospital of Ancona (N. 2016-0424, approval date: 20 October 2016).

### 5.5. In Vitro sIgE Reactivity

Experimental ImmunoCAP tests with Ves v 2.0101 were conducted as outlined by Marknell et al. [36]. Sensitization rates for recombinant Api m 2, Pol d 2, and Ves v 2.0201 were assessed in vitro using ELISA. Purified proteins (40 µg/mL) were coated onto 384-well microtiter plates (Nunc, Thermo Fisher Scientific, Ulm, Germany) and incubated overnight at 4 °C. The remaining binding sites were blocked with 40 mg/mL fat-free milk powder in PBS at room temperature for 1 h. Negative controls (wells without recombinant hyaluronidase) were included. Patient sera were diluted with two parts PBS, added to the wells, incubated overnight at 4 °C, and washed four times with PBS. Bound sIgE was detected using a monoclonal anti-human IgE antibody conjugated to alkaline phosphatase (BD Pharmingen, Heidelberg, Germany) at a 1:1000 dilution.

After a second washing step, a substrate solution (100 mM Tris, 10 mM MgCl_2_·6H_2_O, 100 mM NaCl, pH 9.5, with 5 mg/mL 4-nitrophenylphosphate; AppliChem, Darmstadt, Germany) was added, and absorbance was measured at 405 nm. Two independent experiments were pooled to generate a single dataset, and mean values were used to calculate sIgE reactivity. Sensitization status (positive vs. negative) was determined using an established cut-off calculation method [37,38,39], assessed per plate, protein, and patient group:

(1) $$\left(\bar{nc}+3\times {SD}_{nc}\right)\times 1.1$$

with $\bar{nc}$ representing the mean absorbance of the negative controls and ${SD}_{nc}$ the standard deviation of this mean. To avoid systematic overestimation of sensitization rates, 10% was added to the calculated cut-off value. In the graphs, this cut-off is depicted as a dotted line. A range within 5 percentage points is provided in the main text instead of the exact calculated value.

### 5.6. Basophil Activation Tests

Basophil activation tests (Flow CAST, Bühlmann Laboratories AG, Schönenbuch, Switzerland) were performed as previously described [40]. Briefly, venous blood from patients was collected in EDTA tubes (1.6 mg EDTA/mL blood). Recombinant proteins were dissolved in PBS and diluted in stimulation buffer to final concentrations of 1.6, 8, 40, 200, and 1000 ng/mL. Stimulation buffer alone served as the negative control, while a monoclonal anti-FcεRI antibody was used as the positive control. A volume of 50 µL venous blood was combined with 20 µL staining reagent (anti-CD63-fluorescein isothiocyanate and anti-CCR3-phycoerythrin monoclonal antibodies) and 100 µL stimulation buffer containing calcium, heparin, and Il-3 (2 ng/mL). This mixture was added to the antigen dilution series and incubated at 37 °C for 25 min. To stop stimulation, 2 mL lysis buffer was added and incubated for 5 min at room temperature. Samples were then centrifuged at 500 rcf for 5 min, and the supernatant was replaced with 300 µL washing buffer before analysis by flow cytometry. Basophils were gated from the lymphocyte population using anti-CCR3. Typically, between 400 and 500 basophilic cells (minimum of 300) were analyzed to assess CD63 upregulation upon stimulation. The percentage of CD63-positive basophils within the total basophil population was used to measure activation (Figure S1). A 10% cut-off, as recommended by the manufacturer, was applied and is represented as a dotted line in the graphs.

## Figures and Tables

**Table 1:** Prevalence of sensitization to venom hyaluronidases.

	Sensitization in %				
	HBV	PDV	YJV	PDV/YJV	HBV/PDV/YJV
Api m 2	45	25	17	17	25
Pol d 2	14	25	17	17	17
Ves v 2.0201	17	6	17	8	25

Sensitization to recombinant Api m 2, Pol d 2, and Ves v 2.0201 in HBV (mono)-, PDV (mono)-, YJV (mono)-, PDV/YJV double-, and HBV/PDV/YJV triple-reactive patients was determined by ELISA. HBV, honey bee venom; mono, mono-reactive in skin test; PDV, *Polistes dominula* venom; YJV, yellow jacket venom.

## Data Availability

The original contributions presented in the study are included in this article and Supplementary Material. The raw data supporting the conclusions of this article will be made available by the authors, without undue reservation.

## Supplementary Materials

The following supporting information can be downloaded at https://www.mdpi.com/article/10.3390/toxins16110498/s1, Figure S1: Flow cytometry graphical analysis for detecting CD63^+^ basophils. Table S1: Clinical patient data.
