# Supplementary material for: Comparative Assessment of the Allergenicity of Hyaluronidases from Polistes dominula (Pol d 2), Vespula vulgaris (Ves v 2), and Apis mellifera Venom (Api m 2)
Source: Toxins (Basel). 2024 Nov 19;16(11):498. doi: 10.3390/toxins16110498 (PMC11598713; doi:10.3390/toxins16110498)
Supplement: Supplementary file 1 [file toxins-16-00498-s001.zip › Table S1.pdf]

Table S1. Clinical patient data.

A '+' indicates a positive test result, and a '–' indicates a negative test result. HBV, honey bee venom; i.c., intracutaneous skin test; PDV, *Polistes dominula* venom; sIgE, specific IgE; tIgE, total IgE; YJV, yellow jacket venom

Patient ID	SPT HBV	SPT YJV	i.c. HBV	i.c. YJV	i.c. PDV	tIgE [kU/L]	sIgE HBV [kU$_A$/L]	sIgE Api m 1 [kU$_A$/L]	sIgE Api m 2 [kU$_A$/L]	sIgE Api m 3 [kU$_A$/L]	sIgE Api m 5 [kU$_A$/L]	sIgE Api m 10 [kU$_A$/L]	sIgE YJV [kU$_A$/L]	sIgE Ves v 1 [kU$_A$/L]	sIgE Ves v 5 [kU$_A$/L]	sIgE PDV [kU$_A$/L]	sIgE Pol d 5 [kU$_A$/L]	Tryptase [ng/mL]
**Patients Germany: Sera used for ImmunoCAP tests with Ves v 2.0101**																		
**HBV (mono) – skin test mono-positive with HBV**																		
33551	+	-		-		83.6	9.3						0.01	0	0.02			4.22
33619	+	-				80.2	6.54						0.08	0	0.01			3.26
33887	-	-	+	-		101	0.5						0.08	0.02	0.25			5.51
34012	-	-	+	-		214	53.8						0.15	0	0.02			2.9
34416	+	-		-		34.3	1.5						0.05	0	0.02			5.41
35277	-	-	+	-		167	2.87						0	0.06	0.15			3.86
36734	+	-		-		14.2	5.26						0.06	0.04	0.16			2.9
37213	+	-		-		11.6	5						0	0	0.01			2.33
37695	-	-	+	-		20.6	1.06						0.11	0	0.01			5.28
38762	+	-		-		53.8	0.95						0.18	0.22	0.18			2.33
39591	+	-		-		50.5	1.53						0.03	0.02	0.07			3.53
41392	-	-	+	-		28.8	0.6						0.03	0.04	0.09			5.41
41718	-	-	+	-		61.6	6.89						0.04	0.01	0.05			9.41
42280	-	-	+	-		29.5	17.1						0	0	0			7.57
42426	-	-	+	-		14	5.34						0.1	0	0			6.76
42439	+	-		-		17	0.71						0.03	0	0.02			3.07
39917	+	-		-		39.7	1.43						0.19	0.07	0.07			2.67
41859	-	-	+	-		15.2	2.95						0.15	0	0.03			<1.0
38005	+	-		-		260	25						0.14	0.01	0.13			1.78
38837	+	-		-		27	14.7						0.27	0	0.37			5.27
38906	+	-		-		27.2	9.15						0.07	0.01	0.03			3.56
39187	+	-		-		107	19.9						0.07	0.01	0.02			3.46
37723	+	-		-		11	0.51						0.06	0.02	0.01			5.7
38677	-	-	+	-		33.5	4.48						0.45	0.14	0.02			3.84
39029	+	-		-		95.5	33.3						0.34	0.01	0.08			6.75
40290	-	-	+	-		34.2	1.67						0.16	0.01	0.26			6.61
36222	+	-		-		41.8	10.5						0.24	0.03	0.06			5.33
37533	+	-		-		94.7	5.03						0.17	0.01	0.03			3.97
33480	-	-	+	-		162	8.84						0.81	0.08	1.31			16.9
38682	+	-		-			20.6						0.4	0.01	0.02			3.33
38427	+	-		-		23.5	1.13						0.77	0.32	0.09			3.33
41421	+	-				61.9	9.51						0.07	0	0			6.77
33219	-	-	+	-		73.2	60.7						0.76	0.24	0.02			4.65
37631	+	-		-		315	50.2						4.96	0.15	0.12			3.2
38622	+	-		-		168	83.5						37.7	1.88	2.45			3.07
**YJV (mono) – skin test mono-positive with YJV**																		
31928	-	+	-			77	0.05						0.36	0.11	0.71			2.6
32021	-	+	-			31.8	0.05						0.38	0.04	0.56			1.97
33593	-	+	-			73.2	0.02						1.07	0.03	9.74			2.78
33638	-	-	-	+		85.2	0.01						4.34	0.02	25.8			6.54
33701	-	+	-			135	0.38						15.5	17.7	3.94			4.56

33897	-	+	-			18.6	0.11						5.64	5.24	2.05			4.37
34009	-	-	-	+		31.8	0.04						1.38	1.08	0.07			3.1
34123	-	-	-	+		21.1	0.02						0.6	0	0.53			4.22
34184	-	-	-	+		21	0.04						1.82	0.53	2.86			2.27
34290	-	-	-	+		19.8	0.04						0.65	0.64	1.33			4.18
34300	-	-	-	+		55.1	0.24						4.84	6.19	0.61			3.57
34360	-	+	-			31.8	0.02						0.75	0	1.19			1.05
34559	-	-	-	+		266	0.08						5.96	0.75	28.5			2.6
34616	-	-	-	+		266	0.09						0.64	0.09	1			3.73
34667	-	+	-			178	0.06						0.71	0.02	34.8			4.03
34745	-	-	-	+		50.1	0.03						1.03	0	2.5			4.43
35459	-	+	-	+		102	0.04						0.55	0.01	1.49			5.76
35930	-	+	-			19	0.04						1.22	0	3.43			6.57
35971	-	+	-			76.9	0.05						1.13	0.01	0.38			3.34
36092	-	-	-	+		24.3	0.09						2.3	0.84	0.39			6.53
36504	-	+	-			14.5	0.13						1.15	0	1.57			3.36
36881	-	+	-			42.5	0.02						7.12	3.41	5.8			3.19
37273	-	+	-			72.2	0.02						1.13	0.34	14.9			3.02
38268	-	+	-			34.9	0.05						0.13	0	0.03			10.4
38506	-	-	-	+		32	0.06						2.69	2.55	19			4.7
38683	-	-	-	+			0.65						11.4	0.04	60.8			2.63
39030	-	+	-			53	0.07						1.39	0.11	4.53			4.78
39386	-	+	-			49.1	0.46						1.67	0	4.44			5.66
39606	-	+	-	+		64.8	0.04						0.4	0	1			3.4
39653	-	-	-	+		49.8	0.1						8.93	40.3	6.26			3.13
39854	-	+	-			44.4	0.6						6.59	7.76	2.07			2.71
39893	-	-	-	+		117	10.6						0.7	1.25	2.99			3.46
40346	-	+	-			114	0.09						0.67	0.96	0.5			4.01
40362	-	-	-	+		36.5	0.04						0.27	0.02	1.09			5.46
40502	-	-	-	+		44	0.2						0.85	0.02	2.54			7.09
40854	-	-	-	+		22	0.02						6.78	6.02	2.39			2.9
41612	-	-	-	+		125	0.04						6.91	0	0.31			2.35
41678	-	+	-			198	0.15						16.9	12	38.6			5.6
41892	-	+	-			47.3	0.21						4.73	0.01	7.53			4.67
41895	-	-	-	+		56.6	2.09						2.05	0.65	11.5			5.37
36253	-	-	-	+		89.3	0.11						3.87	1.07	9.48			1.5
36835	-	-	-	+		65.2	0.46						2.62	0.3	19			3.86
37298	-	+	-			115	0.1						5.01	2.8	14.6			4.06
41118	-	-	-	+		29.9	0.01						1.12	0.58	0.82			4.21
42008	-	-	-	+		64.2	0.16						1.09	0.11	1.71			3.32
42212	-	-	-	+		490	0.25						2.78	1.52	0.19			3.81
42253	-	-	-	+		126	2.86						4.12	4.05	0.36			15.7
42661		+				39.4	0.32						1.36	0.26	11.3			4.59
36351	-	+	-			56.2	0.27						2.87	0.04	9.1			2.83
38346	-	+	-			46	0.36						1.05	0.08	3.2			23.4
39028	-	-	-	+		197	0.23						1.56	1.12	7.48			8.9
40930	-	+	-			920	2.17						1.92	0.03	23.7			5.55
41818	-	-	-	+		370	0.14						10.2	6.1	52.9			4.78
37401	-	+	-			341	0.57						9.01	4.6	3.79			5.18
38197	-	+	-			46.2	0.16						0.53	0.08	2.96			3.58
40204	-	-	-	+		53.1	0.23						3.9	3.9	3.85			2.32
41497	-	-	-	+		468	1.07						15	1.09	29.2			9.1
38748	-	-	-	+		5.9	0.36						0.55	0.61	0.02			5.3
38962	-	-	-	+		17	0.63						7.73	9.36	0.02			5.03
41906	-	-	-	+		499	1.14						45.1	1.25	>100			4.81
33049	-	-	-	+		117	0.08						0.74	0.73	0.87			2.46

34302	-	+	-	+		16.3	0.14						0.8	0.27	0.22			3.77
41994	-	-	-	+		56.6	0.54						1.38	0.12	5.39			1.41
42705	-	-	-	+		21.8	0.16						1.29	0.21	2.09			16.7
37296	-	-	-	+		1266	5.28						4.91	2.16	1.95			2.86
40683	-	+				31.9	0.07						2.7	0.3	4.3			3.73
31968	-	+	-			76.6	0.08						0.72	0.45	0.93			3.36
40991		+				62.9	0.54						1.79	0.73	0.5			20.1
39211		+				25.3	0.2						0.95	0.52	0.27			3.12
40015	-	-	-	+		1014	1.95						3.4	0.66	4.79			12.4
42266	-	-	-	+		86.7	0.23						24.7	16.7	3.93			3
32911	-	+	-	+		533	4.12						1.94	0.13	7.52			2.33
41998	-	-	-	+		152	0.94						9.81	6.38	9.5			3.07
35033	-	-	-	+		26.2	0.05						9.9	0.77	0.99			4.66
38755	-	+	-	+		275	4.55						21.1	15.9	2.82			4.22
42420	-	-	-	+		142	0.88						9.69	4.13	12.7			3.27
41965	-	+	-			37.1	0.03						0.69	0.23	4.81			3.14
33435	-	+	-	+		315	1.74						8.55	0.15	15.8			8.28
**HBV / YJV – skin test double-positive with HBV and YJV**																		
41748	-	-	+	+		90.4	2.97						3.45	1.04	4.38			5.62
41379	-	-	+	+		241	1.02						4.39	3.55	7.42			14.8
40282	-	-	+	+		26	0.23						0.56	0.38	0.24			4.14
38660	+	+				127	0.44						9.07	2.37	12.7			6.83
40598	-	-	+	+		89	1.85						2.03	0.07	9.25			6.38
33334	-	-	+	+		126	3.9						16.2	18.8	8.53			3.57
41886	-	-	+	+		18.1	3.25						12	1.43	38.2			<1.00
38692	+	-		+		212	2.07						3.59	0.77	4.5			4.92
42407	+	+	+	+		1589	27.7						51.4	32.8	32.8			4.04
34265	-	-	+	+		415	21.1						12.7	0.28	18.9			3.75
38881	-	+	+			1191	18.7						55.2	45.7	12.1			2.56
34558	+	+				166	14.1						56.8	41.3	22.9			3.86
38900	-	+	+			64	0.51						4.83	0.23	10.3			8.91
40579	-	-	+	+		39.5	1.04						2.63	1.22	3.42			3.4
42392	+	+				394	31.7						0.54	0.02	0.09			2.7
32219	-	-	+	+		36.3	0.91						0.46	0.05	0.84			4.58
39949	-	-	+	+		18.5	0.04						0.54	0.17	0.05			2.59
40273	+	+	+	+		84.9	0.76						2.06	0.03	4.17			4.29
40853	-	-	+	+		76.4	2.84						7.68	1.2	42.2			<1.00
37190	+	+				167	0.52						6.63	1.72	19.4			5.83
32209	-	-	+	+		29.8	2.69						0.59	0.77	0.08			11.6
34855	+	+				107	1.22						1.94	1.19	7.64			8.9
41583	-	-	+	+		30.5	1.09						0.16	0.01	0.22			4.42
42205	-	-	+	+		567	3.9						0.6	0.01	0.95			2.1
34376	+	+				45.4	3.42						0.7	0.17	1.06			4.9
34168	+	-	+	+		105	34.4						2.43	0.22	3.57			2.83
37191	-	+	+			324	28.2						3.92	1.73	1.5			3.53
37652	+	+				47.2	1.12						1.12	0.3	1.03			3.21
37587	-	-	+	+		91	37.9						9.41	0.12	0.27			1.64
34574	+	+				49.3	8						4.06	3.79	0.66			13.1
**Patients Italy: Sera used for *in vitro* assessment of sensitization rates to Api m 2, Pol d 2, and Ves v 2.0201**																		
**PDV (mono) – skin test mono-positive with PDV**																		
1			-	-	+	520	0.22						1.47	1.17	2.63	5.36	0.01	6.8
2			-	-	+	58.30	0.08	0.00					1.78	1.78	0.03	16.50	0.24	4.1
3			-	-	+	65.10	0.03	0.00					0.15	0.04	0.03	4.64	0.01	4.3
4			-	-	+	45.2	0.04	0.02					0.06	0.03	0.03	2.22	0.43	7.0
5			-	-	+	31.2	0.04						0.03	0.00	0.00	0.91	0.43	5.7
6			-	-	+	78.6	0.26	0.00				0.00	0.82	0.13	0.00	3.58	0.18	10.3

7			-	-	+		0.30	0.00				0.00	0.54	0.08	0.58	3.21	1.78	2.6
8			-	-	+	16.4	0.51	0.02				0.01	0.65	0.07	1.08	1.05	1.00	2.8
9			-	-	+	50.0	0.01	0.00					1.48	1.71	0.07	3.55	0.06	3.3
10			-	-	+	45.2	0.14	0.00					0.44	0.05	0.09	0.93	0.14	11.1
11			-	-	+	220	0.09	0.01					2.74	3.36	0.48	8.25	0.94	3.7
12			-	-	+	30.5	0.02						0.00	0.00	0.00	1.78	0.07	32.1
13			-	-	+	20.3	0.03						0.10	0.00	0.08	4.62	1.15	2.0
14			-	-	+	60.9	0.03						0.03	0.00	0.09	1.31	1.31	4.5
15			-	-	+	29.4	0.01						0.09	0.09	0.00	0.33	0.00	4.2
16			-	-	+		0.07						0.08	0.00	0.15	5.23	5.41	3.4
**YJV (mono) – skin test mono-positive with YJV**																		
17			-	+	-	356	0.12	0.01					0.91	0.01	0.36	0.06	0.07	4.8
18			-	+	-	31.2	0.02						2.70	1.15	0.68	0.12	0.01	5.0
19			-	+	-	301	0.98	0.0					3.03	0.49	2.42	0.62	0.56	10.9
20			-	+	-	29.8	0.01						0.23	0.10	0.13	0.02	0.00	5.1
21			-	+	-	58.3	0.03						11.50	12.80	0.37	2.66	0.03	4.5
22			-	+	-	163	0.10	0.03					1.14	0.20	0.82	0.01	0.00	6.6
23			-	+	-	27	0.06						0.85	0.00	0.81	0.14	0.10	5.4
24			-	+	-	49.1	0.70	0.00					16.50	14.60	1.20	0.10	0.09	3.6
25			-	+	-	127	0.00						1.24	0.00	1.82	1.68	2.19	3.7
26			-	+	-	108	0.01	0.00					5.04	0.04	4.77	0.24	0.95	4.9
27			-	+	-	173	0.05						0.18	0.00	0.19	0.90	0.00	7.7
28			-	+	-	160	0.01						2.70	3.35	0.11	0.15	0.00	4.4
29			-	+	-	70.1	0.17	0.00					4.39	0.10	3.50	0.10	0.10	3.9
30			-	+	-	20.6	0.03						1.56	2.75	0.06	0.03	0.00	4.3
31			-	+	-	219	0.08						9.08	0.43	12.60	1.10	0.10	6.9
32			-	+	-	22.3	0.06						2.13	1.53	0.71	0.09	0.09	5.3
33			-	+	-	19.1	0.02						0.58	0.11	0.30	0.06	0.00	3.9
34			-	+	-	27.3	0.70	0.00					1.05	0.04	0.74	0.03	0.00	5.4
**HBV (mono) – skin test mono-positive with HBV**																		
35			+	-	-	59.3	0.53	0.00	0.0		0.00	0.79	0.03		0.00	0.04	0.00	4.6
36			+	-	-		13.50	6.06	0.01	0.64	0.18	4.34	0.16		0.04	0.94	0.04	20.1
37			+	-	-	46.1	1.49	0.07	1.37	0.00	0.21	0.01	0.00	0.02	0.00	0.00	0.00	8.30
38			+	-	-	108	8.95	4.09	1.68		0.02	0.40						8.1
39			+	-	-	46.9	28.90	17.30	0.03	3.34	2.08	8.30	0.00		0.00	0.00	0.00	5
40			+	-	-	162	25.50	4.08	13.40		4.37	18.30	1.61	0.90	0.46	0.70	0.28	4.2
41			+	-	-	37.9	1.17	0.94	0.08	0.32	0.00	0.02			0.00		0.00	12.2
42			+	-	-	48.4	16.60	3.37				1.80						4.0
43			+	-	-	19.7	7.35	6.90	0.00		0.00	0.04	0.01			0.01		3.6
44			+	-	-	34.7	4.36	2.07	0.00		0.25	0.02	0.17	0.02	0.00	0.17	0.00	3.8
45			+	-	-	193	67.70	9.69				3.91	0.31			0.10		5.1
46			+	-	-	203	58.30	17.90				9.74	1.11	0.13	0.18	0.85	0.39	2.4
47			+	-	-	58.2	4.95	0.61				2.02	1.07			1.03		1.5
48			+	-	-	46.2	5.43	0.31	2.37	0.20	0.40	3.09	0.08	0.02	0.09	0.96	0.79	12.4
49			+	-	-	120	10.30	8.20	7.37	0.03	0.01	0.04	0.01			0.01		6.8
50			+	-	-	115	8.70	3.79				0.05	0.21			0.10		6.3
51			+			54.7	34.80	27.00	2.20	0.02	1.60	0.62	0.15			0.13		3.2
52			+	-	-	30.3	2.33	0.50					0.04			0.16		5.0
53			+	-	-	247	29.90	2.07					0.24			0.68		3.5
54			+			169	62.20	62.30					0.12			0.18		4.7
55			+	-	-	240	14.80	0.94					0.19			0.30		6.6
56			+	-	-	599	1.98	0.21										3.5
57			+	-	-	78.2	17.30	5.39					0.07		0.000	0.70	0.00	1.4
58			+	-	-	210	4.37	0.08										6.1
59			+	-	-	137	15.50	4.22					0.13	0.010	0.120	0.03	0.09	3.5
60			+	-	-	34.1	0.71	0.56					0.01			0.01		7.1

61			+	-	-	89.5	2.78	0.19					0.01			0.01		2.7
62			+	-	-	57.5	0.41	0.32					0.05			0.03		5.7
63			+	-	-	54.6	1.09	0.46					0.03			0.04	0.00	4.8
**PDV / YJV – skin test double-positive with PDV and YJV**																		
64			-	+	+	12.8	0.09						1.94	0.33	2.04	0.67	0.93	4.7
65			-	+	+	985	0.80	0.01					69.30	80.10	22.70	56.10	20.10	15.4
66			-	+	+	98	0.02						2.29	0.03	3.02	2.23	2.77	3.8
67			-	+	+		10.00	0.10					>100	64.10	61.10	>100	75.50	2.6
68			-	+	+	367	0.10	0.01					44.30	14.70	51.00	11.30	16.10	4.2
69			-	+	+		0.51	0.03				0.35	5.58	6.63	0.62	>100	3.49	2.3
70			-	+	+	115	0.04	0.00					6.65	4.82	2.50	3.69	1.78	7.0
71			-	+	+	56.5	0.02	0.00					3.20	1.22	1.53	1.42	2.03	6.9
72			-	+	+		0.78	0.01					18.80	0.06	10.60	27.70	22.40	2.8
73			-	+	+	110	0.21	0.01					9.00	1.53	5.71	3.28	0.99	3.4
74			-	+	+		0.10	0.04					32.20	2.39	27.90	21.60	29.80	7.9
75			-	+	+	89.8	0.03	0.01					5.51	5.81	0.16	7.70	0.94	6.3
76			-	+	+	377	0.76	0.02					3.48	0.54	2.96	2.46	2.34	5.6
77			-	+	+	166	0.06	0.01					6.20	6.56	0.59	6.15	1.39	5.1
78			-	+	+	1798	0.21	0.10					85.00	33.80	87.90	22.20	37.90	3.1
79			-	+	+	19	0.03						0.69	0.45	0.23	0.19	0.06	4.3
80			-	+	+	110	0.26	0.01				0.05	4.05	4.65	0.29	1.31	0.21	5.3
81			-	+	+	988	3.71	0.01					40.90	24.90	31.50	93.60	60.30	5.5
82			-	+	+	431	1.66	0.13					>100	3.20	25.20	11.60	6.06	2.3
83			-	+	+	312	0.05	0.00					49.90	25.10	53.10	>100	>100	1.5
84			-	+	+	105	1.01	0.12					6.22	3.09	2.35	6.66	2.88	2.9
85			-	+	+	109	0.88	0.10					3.86	2.33	1.52	4.59	1.60	5.4
86			-	+	+	308	0.40	0.10					93.30	67.60	40.70	>100	76.60	4.6
**HBV / PDV / YJV – skin test triple-positive with HBV, PDV, and YJV**																		
87			+	+	+	245	2.070	0.070					11.900	2.860	7.050	7.090	5.360	4.3
88			+	+	+	1769	1.200						60.000	51.900	14.700	5.650	5.180	3.1
89			+	+	+	280	6.100	0.360			1.640	0.320	>100	0.570	>100	93.900	>100	7.0
90			+	+	+	492	2.790	1.010					5.570	3.220	4.220	9.290	6.340	4.0
91			+	+	+	123	10.800	0.130			2.280	0.120	14.000	3.570	5.140	14.000	7.270	4.2
92			+	+	+	233	1.020	0.020			0.430	0.060	1.640	0.630	0.090	0.970	0.030	4.7
93			+	+	+	428	2.52	1.20					31.800	0.540	35000	36.800	40.800	4.1
94			+	+	+	22.2	1.290	0.000					8.970	5.410	2.070	1.630	0.490	3.9
**Patients Germany: Blood samples used for basophil activation tests**																		
M1				+		24.3	<0.10	<0.10		<0.10	<0.10	<0.10	2.11	<0.10	1.51			19
M2			+	+		36	1.63	<0.10		<0.10	<0.10	<0.10	2.78	<0.10	0.8			5.29
M3			-	+				<0.10	<0.10	<0.10	<0.10	<0.10	1.30	<0.10	9.31			
M4			+	+		73.5	11.0	3.15		1.38	7.44	5.01		<0.10	<0.10			3.26
M6				+		14.4	26.4	9.69	0.9	1.67	<0.10	13.7	2.37	<0.10	2.37			36.6
M8			-	+		61.7												3.14
M9			-	+		128	<0.10	<0.10	<0.10	<0.10	<0.10	<0.10	14.2	<0.10	89.7			6.31
M10			-	+		934	1.86	<0.10	<0.10	<0.10	<0.10	<0.10	16.2	<0.10	16.9			4.41
M11			-	+			<0.10	<0.10		<0.10		<0.10						
M12			-	+		90.5	<0.10	<0.10	<0.10	<0.10	<0.10	<0.10	13.2	7.55	7.38			3.35
M13			+	-		103				<0.10		<0.10	<0.10					4.06
M14			+	-		146												2.81
M15						20.4												6.58
M16			+	-		77.1												
M18			+	+		144	8.38	0.69	6.91	0.19	9.71	0.34	2.61	1.06	0.13			
M19			+	-		93.8	>100	37.3	18.7	1.43		1.28	0.463	<0.1				2.57
M21			+	-		127	> 100	34.7		12.7	8.72	2.54		0.72	0.18			5.51
**PatientsSpain: Blood samples used for basophil activation tests**																		
B1			-	-	+	96.5	0.13	0.00					11.6	14.3	0.23	22.1	5.62	4.63

B3			-	+	+	13.7	0.00	0.00					3.20	3.09	0.21	3.25	0.08	7.33
B4			+	+	+	144	10.9	5.39	1.00	0.05	4.54	0.78	4.11	2.36	0.64	4.44	0.79	
B6			+	-	-	11	0.94	0.54	0.00	0.07	0.00	0.43	0.01	0.02	0.00	0.00	0.00	
B7			+	-	-	94.1	1.75	1.07	0.32	0.01	0.12	0.04	0.31	0.05	0.00	0.38	0.14	
B8			-	+	+	427	0.01	0.00	0.05	0.00	0.01	0.01	51.4	0.97	>100	>100	>100	
B9			-	-	-	40.5	0.04						1.21	0.92	0.13	1.65	0.55	
B10			-	-	+	311	0.04	0.01					1.56	0.28	2.11	9.11	6.77	3.82
B11			-	+	+	237	0.02	0.00					10.6	0.03	23.1		36.1	3.67
B12			-	+	+	378	0.00	0.00					3.81	0.15	4.68		6.94	2.69
B13			-	+	+	102	0.00						0.94	1.02	0.15	2.53	0.9.	3.68
B14			-	+	+	21.9	0.00						1.77	0.01	1.88	1.62	1.28	3.21
