# Supplementary material for: Comparative Assessment of the Allergenicity of Hyaluronidases from Polistes dominula (Pol d 2), Vespula vulgaris (Ves v 2), and Apis mellifera Venom (Api m 2)
Source: Toxins (Basel). 2024 Nov 19;16(11):498. doi: 10.3390/toxins16110498 (PMC11598713; doi:10.3390/toxins16110498)

Figure S1. Flow cytometry graphical analysis for detecting CD63^{+} basophils.

A dot plot 1 was created as Forward Scatter vs Side Scatter to acquire the whole leukocyte population (A). Dot plot 2 was created as CCR3-PE vs. Side Scatter. A gate including the entire basophil population as CCR3-positive and SSC-low was set (B). Dot plot 3 was created as CD63-FITC vs CCR3-PE showing only the gated basophils. The non-stimulated, resting basophils (background) are used to set a quadrant gate including CD63-negative basophils in the lower right quadrant. Activated basophils resulted in a CD63-positive basophil population identified in the upper right quadrant (C). The readout of the assay is indicated as the ratio of CD63-positive basophils over all basophils (%CD63 activation).

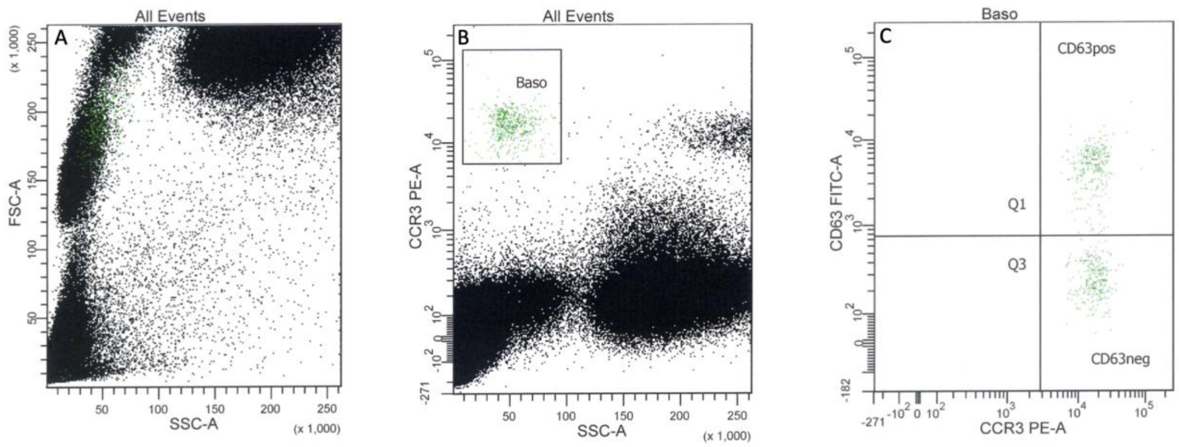

Supplement: Supplementary file 1 [file toxins-16-00498-s001.zip › Figure S1.pdf]
